# Brain Cortex Activity in Children With Anterior Open Bite: A Pilot Study

**DOI:** 10.3389/fnhum.2020.00220

**Published:** 2020-06-30

**Authors:** Claudia Restrepo, Paola Botero, David Valderrama, Kelly Jimenez, Rubén Manrique

**Affiliations:** ^1^CES-LPH Research Group, Universidad CES, Medellín, Colombia; ^2^GIOM Group, Universidad Cooperativa de Colombia, Envigado, Colombia; ^3^Visión Integral, Medellín, Colombia

**Keywords:** brain cortex, anterior open bite, children, deglutition, phonation

## Abstract

Anterior open bite (AOB) is related to functional alterations of the stomatognathic system. There are no studies concerning brain activation of the cortex comparing children with and without AOB during rest and activities such as deglutition and phonation. The aim of this study was to determine the activity of the brain cortex of children with AOB at rest and during phonation and deglutition and to evaluate the association of intelligence quotient (IQ), attention (Test of Variables of Attention, known as TOVA), beats per minute (BPM), and oxygen saturation measurement (SpO_2_) with brain activity in subjects with AOB. Fourteen children (seven with AOB and seven without AOB) with mixed dentition, aged 10–13 years, underwent an IQ test, TOVA, SpO_2_, and quantitative electroencephalography (QEEG). Electrodes were set in the scalp, according to the 10–20 protocol. Data were analyzed using statistical tests to assess comparisons between children with and without AOB. The results showed that IQ, TOVA, SpO_2_, or BPM did not show any statistically significant differences between the groups, except for the response time (contained in TOVA) (*p* = 0.03). Significant differences were found for the brain activity during rest (Condition 1) of the tongue, between children with and without AOB (*p* < 0.05 for alpha/theta and alpha peaks), whereas there were no differences during function (Condition 2). The findings of this investigation provide insights about the cortex activity of the brain while the tongue is in the resting position in children with AOB. This may imply an altered activity of the brain cortex, which should be considered when diagnosing and treating AOB. Other diagnostic techniques derived from investigations based on neuroscience could develop new diagnostic and therapeutic techniques to give better solutions to children with malocclusions. Treatments should be focused not only on the teeth but also on the brain cortex.

## Introduction

Anterior open bite (AOB) is defined as the absence of vertical overlap between the maxillary and mandibular incisors (Subtelny and Sakuda, 1964). Its prevalence is about 2.7% in children between 8 and 16 years of age (Ocampo-Parra et al., 2015). It has a multifactorial etiology. The improper posture of the tongue at rest (Knösel et al., 2015) and its size and function (Zhou et al., 2016), as well as oral habits (Chen et al., 2015), neurological disturbances (Martinez-Mihi et al., 2014), and airway obstruction (Cazzolla et al., 2010), play a significant role in the origin of AOB.

It is well established that many functions of the tongue, such as deglutition and phonation (Ludlow, 2015; Xiao et al., 2017), depend on brain cortex regulation. Brain cortical representation of these functions has previously been investigated (Kober et al., 2015). It is well established that the central sulcus area, in which the ventral half of the sensorimotor cortex is located, is related to the tongue’s functions of deglutition, movement, and coordinated movements of phonation (Breshears et al., 2015). These processes are influenced by cognitive performance (Burgaleta et al., 2014) and oxygen saturation (Rodriguez Moreno et al., 2015).

Previous investigations indicate that AOB is present in around 13% of mouth breathers (Pacheco et al., 2015). Electroencephalography (EEG) signals in mouth breathers present a brain activity involving lower theta and alpha powers at rest when compared with that of nose breathers. This activity comprises cognitive regions and involves decreased oxygen saturation during mouth breathing. This issue suggests that when cognition is required, mouth breathing can act as one of the variables that could cause alteration in brain function, especially in memory tasks (Lee et al., 2019).

Studies concerning brain activation when the tongue is at rest and during deglutition and phonation in children with AOB are not available and could be the answer to why orthodontic treatments of AOB are so unstable (Bondemark et al., 2007). To avoid a relapse of AOB treatments, changing the motor response must be permanently imprinted in the brain (trained) (Svensson et al., 2003). The learning process occurs because a movement that has been elicited repeatedly by successive stimuli may, after a while, be evoked without the need of the conditioning stimulus, because it has been imprinted in the cerebral cortex (Svensson et al., 2003). Based on this concept, other diagnostic techniques derived from neuroscience investigations could develop new diagnostic and therapeutic techniques to give better solutions to children with malocclusions. The approach from neuroscience in the case of AOB could give support as to why treatments relapse is less when myofunctional therapy is used than when only orthodontics is performed (Smithpeter and Covell, 2010).

According to the above statements, the aims of this study were to (1) determine the activity of the brain cortex of children with AOB at rest and during phonation and deglutition and (2) to evaluate the association of intelligence quotient (IQ), attention [Test of Variables of Attention (TOVA)], and oxygen saturation with brain activity in subjects with AOB.

## Materials and Methods

### Approval and Design

This investigation was approved by the ethics committee of Universidad CES (file number 60-225). The children and their parents gave their written informed consent and assent to participate in the study.

**FIGURE 1:**
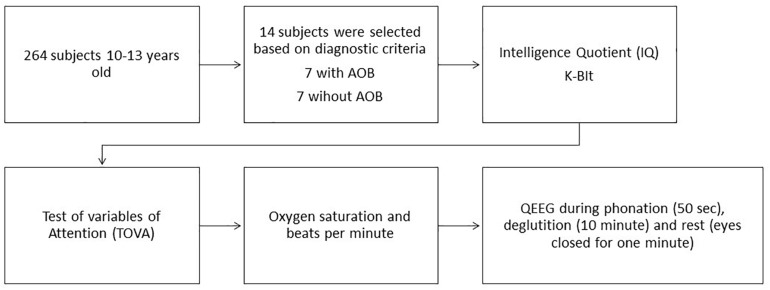
Measurement sequence and procedures.

The study was performed according to an exploratory design. Sequence and measurement procedures are shown in Figure 1.

### Population and Sample

Children (10–13 years of age) with mixed dentition with fully erupted maxillary and mandibular incisors and with complete posterior occlusion, with either primary or permanent molars or bicuspids, were included. Mixed dentition is defined as the transition stage between primary and permanent dentition, when the first molars erupt and the primary teeth are replaced by permanent teeth (Louly et al., 2011). The sample size was calculated based on the formula by Viechtbauer et al. (2015), assuming the highest difference between brain cortex activity of children with and without AOB. Data collection was performed from June 2015 to December 2015.

Children were selected from a previous study of 264 students, recruited from public schools of Envigado, Colombia (Ocampo-Parra et al., 2015). Subjects in this study were included on the basis of having normal facial morphology (no anatomical abnormalities, such as cleft lip); absence of neurological and/or psychiatric disorders previously diagnosed by a physician and known by the parents (including reading disorders or disabilities); absence of mouth breathing, related by parents and previously diagnosed by a physician; amelogenesis or dentinogenesis imperfecta, present in dental records and/or diagnosed by a pediatric dentist; history of anterior dentoalveolar trauma during the permanent dentition; absence of pretreatment and/or current speech therapy; and absence of previous and/or current preventive or corrective orthodontics or orthopedics. All children were required to present with a Nolla (1960) dental development stage of 10 (apical end of root completed) in permanent incisors and permanent first molars. Finally, 14 children were selected (seven with and seven without AOB).

### Clinical Evaluation of Anterior Open Bite

Subjects included in the study underwent a clinical examination to assess the presence of AOB, sitting in a dental chair, with the same conditions of light. The anterior bite was measured from the upper right central incisor to the lower right central incisor. AOB was considered present, when there was a vertical space >0.1 mm between the maxillary and mandibular teeth, whereas posterior teeth were in occlusion and absent when the vertical space between the maxillary and mandibular incisors was 0 or there was no AOB present (Subtelny and Sakuda, 1964).

### Intelligence Quotient

A Fast IQ test was performed using the K-BIT, which is a Brief Intelligence Kaufman Test^®^ (Kaufman and Kaufman, 2004), which assesses intellectual ability for ages 4–89 years. It evaluates verbal ability (vocabulary and definitions) and non-verbal reasoning ability (matrices). The K-BIT presents internal consistency reliability of 0.90 for verbal scale and 0.86 for non-verbal scale in children aged 4–18 years (Kaufman and Kaufman, 2004; Mervis et al., 2012). Its application takes 15–30 min, and it consists of two subtests:

1. Vocabulary and definitions: A measure of verbal skill that requires oral responses, language development, the formation of verbal concepts, and information flow.
2. Matrices: Measures non-verbal skills and the ability to solve new problems. Evaluates the ability to solve problems of reasoning through visual stimuli both figurative and abstract.

### Test of Variables of Attention

The TOVA is a five-item, self-rated tool that assesses errors of omission (inattention), errors of commission (impulsivity rate), response time, the variability of response time, and double-clicks (error committed when differentiating two simple geometric figures, one of those determined as the target of attention). TOVA was designed for subjects between 8 and 80 years of age, with different scales for each age group. The evaluation takes approximately 22 min (Lawrence et al., 1993).

Attention was measured while responding to a geometric stimulus, which was considered the target of attention. For this investigation “target frequent” was considered when the child focused on the geometric stimuli and “target infrequent” when attention was not paid to the stimuli. The incorrect response to the target was called an “error of commission” or impulsivity, whereas the failure to respond to the designated target was measured as an “error of omission” or inattention. The mean time to respond correctly to the target stimulus and the variability of the response time was considered as “attentional variability.” The multiple and anticipatory responses to the target were designated as double-clicks. TOVA precisely measures reaction times (±1 ms). Its reliability coefficient is 0.84 (Llorente et al., 2001).

### Oxygen Saturation and Beats per Minute

Measurements of oxygen saturation (SpO_2_) and beats per minute (BPM) of the children were performed with a pulse oximeter (Safe Heart FPO40, Beijing Safe Heart Technology, China). SpO_2_ was considered as the measure of the percentage of hemoglobin binding sites in the bloodstream occupied by oxygen (normal SpO_2_ should be between 96 and 99%), whereas BPM was the number of times that the heart beats per minute while it is at rest. Its mean value ranges from 60 to 80.

### Quantitative Electroencephalography

Each child underwent a quantitative EEG (QEEG) at Instituto Psicotecnológico in Medellin, Colombia. QEEG is a validated technique for brain mapping (Thatcher, 2010), which allows highly accurate measurement of the electrical activity of the brain cortex, at rest, and during the execution of specific tasks. The electrical activity was measured with a 2EB Hardware Clinical Brain Master^®^ of Brainmaster System Technologies, Inc. (Ohio, OH, United States). Data were collected using the BioExplorer version 1.6^®^ and BioReview 1.3^®^ software (Cyber Evolution Inc., United States).

Each QEEG examination was performed with the child seated in a right position. Electrodes were set in specific points in the scalp. For that purpose, the scalp was cleaned with Nuprep^®^ skin prepping gel (Weaver and Company, Colorado, CO, United States) to minimize interference with the electrical signals. Afterward, conductive paste (Ten20^®^, Weaver and Company, Colorado, CO, United States) was applied to each electrode. Surface electrodes were positioned on the scalp, following the international 10/20 system and set between the skull points nasion, inion, and preauricular, according to a validated technique (Jurkac et al., 2007).

The central sulcus (referred to herein as the letter C) divides the frontal and parietal lobes of the left and right cerebral hemispheres. The signal from this electrode aimed to measure the primary sensory cortex. The electrode with the even number 4 was positioned in the right hemisphere and the one with the odd number 3, in the left hemisphere (Mervis et al., 2012). In this investigation, only the central sulcus area of the left (C3; Zone 1) and right (C4; Zone 2) hemispheres were analyzed, which are the ones in the brain cortex related to the tongue at rest (Condition 1) and during function (Condition 2) involving deglutition and phonation.

Quantitative electroencephalography recordings were performed in three cases: (1) The children were asked to breathe softly and to close their eyes for 1 min (measurement at rest), and then they were asked to open their eyes for another minute; (2) children were asked to execute a phonation task for 50 s (reading the same text for every child). The reading included dental and alveolar consonants to evaluate the tongue function during phonation. Dental consonants are articulated in the Spanish language with the tongue against the upper teeth, such as /d/, /n/, /t/, and/l/, whereas the alveolars contact the tongue with the gum ridge. (3) Finally, deglutition was recorded for 10 s. Each child was given a glass of water and asked to drink it continuously for 10 s. The test was done with water, to avoid any extrinsic stimulation of salvation.

The QEEG applies mathematical algorithms to transform the data obtained into the following frequency bands: theta/beta, alpha/theta, alpha, beta, and wavelength of 2–38 Hz. The magnitude corresponds to the amount of energy that the original QEEG possesses at each frequency, and it is measured in hertz.

An expert investigator read and analyzed the data and gave a diagnosis based on the guidelines given by the Othmer protocol (Oostenveld and Praamstra, 2001).

### Control Parameters

Both the clinical classification (AOB/No AOB) and the QEEG examination were performed by expert trained investigators. In the previous study from which children were selected for this investigation (Ocampo-Parra et al., 2015), AOB was assessed by two researchers. The consensus was gathered in order to avoid evaluation bias. Kappa index and intraclass correlation coefficient (ICC) were obtained, and the results were 0.95 and 0.99, respectively. For this investigation, the presence or absence of AOB was confirmed before assessing the IQ, attention, oxygen saturation, and QEEG.

Acquiring a QEEG signal properly means avoiding errors and affecting the subject and the biosignal measurements. The goal is to obtain measurements secure for the individual and with high signal-to-noise ratio (SNR) and no data loss. The points that were considered in this investigation were as follows (Usakli, 2010):

1. Subject safety: Leakage currents were avoided by maintaining subjects and front-end circuitry and earth grounds as separate.
2. Electromagnetic interference protection: QEEG signals are distorted, and the signal is corrupted with noise when electrical or electronic devices are near the recording setup. Therefore, operation of those devices closed to the recording setup was prohibited. Additionally, the instrumentation amplifier was used.
3. Subject muscular movements: Muscular movements, different from the ones that were purposed for the investigation, could affect the QEEG signals. Therefore, children were encouraged to avoid those types of muscle activities.
4. Electrostatic discharge (ESD) protection: No ESD protection may cause damage of electronic components and QEEG signals and serious problem for subjects. Thus, active electronic components had greater than 2,000 V of ESD protection.
5. Efficient grounding: The lab where measurements were made had proper grounding techniques to help reduce noise, therefore increasing SNR.
6. Electrodes: Electrodes were dried before setting, and the position was controlled by locating the surface electrodes in a previously calibrated and validated position.
7. Electrode contact impedance: Less than 1 kΩ of contact impedance indicates probable shortcut between the electrodes, and greater than 10 kΩ avoids acquiring EEG signals. Thus, the contact impedance value for electrodes was between 1 and 10 kΩ.
8. Digitization: Sufficient and optimal digital resolution was provided for analog-to-digital conversion in order to impede an increase in the quantization error.
9. Sampling instants: In a multichannel system, the time delay between channels could be a problem. This problem was controlled by using a digital multiplier.

All the QEEG interpretations were made by the same investigator who was blind to the AOB classification.

### Statistical Analysis

A comparison of variables between the anterior AOB and No AOB group was performed, using Mann–Whitney *U* test or *T*-test, depending on the distribution of the variables.

Statistical analysis was performed using STATA version 14.0 (StataCorp LP, Texas, TX, United States).

## Results

**FIGURE 2:**
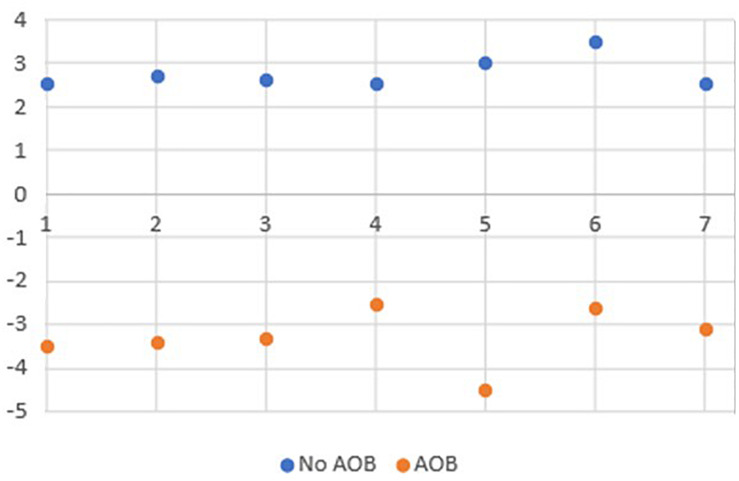
Distribution of children according to vertical space (mm) between the maxillary and mandibular incisors.

This study included 14 child subjects (seven females and seven males; mean age 11.9 years; range 10–13 years old). The children with AOB presented with an open bite with a mean of 3.3 mm (SD 1.3). The distribution of children, according to vertical space (mm) between the maxillary and mandibular incisors, is presented in Figure 2.

When analyzing IQ, TOVA (response time, variability, inattention, commission, double-click), SpO_2_ (mean 97%, SD 1.0), and BPM (mean 73, SD 2.8), no statistically significant differences were found between the groups with and without AOB, except for response time, which presents statistically significant differences when comparing both groups (Table 1).

**TABLE 1:** Comparison of IQ, TOVA, SpO_2_, and BPM between children with and without anterior open bite (AOB).

Variable	Anterior open bite	No anterior open bite	*p-*Value
	Rank sum	Rank sum	
IQ	65	40	0.11
Response time	36	69	0.03
Variability	66.5	38.5	0.07
Inattention	65	40	0.11
Commission	54.5	50.5	0.80
Double-click	59.5	45.5	0.14
Oxygen saturation	57	48	0.56
BPM	57.5	47.5	0.52

*All *p-*values were obtained with Student *T*-test. IQ, intelligence quotient; TOVA, Test of Variables of Attention; SpO_2_, oxygen saturation measurement; BPM, beats per minute.*

When evaluating the brain activity, there are statistically significant differences in the variables frequency alpha/theta during rest in the central sulcus area of the left hemisphere (C3; Condition 1/Zone 1) (*p* = 0.047) and frequency alpha peak during rest in the central sulcus area of the left hemisphere (C3; Condition 1/Zone 1) (*p* = 0.018). The brain activity is higher in subjects with AOB than in subjects without AOB (Table 2). Comparing the frequency alpha/theta with alpha peak during rest in the central sulcus area of the left hemisphere (C3) within the groups, there are no differences between the AOB and control groups (Table 2).

**TABLE 2:** Comparison of cortex activity between children with and without anterior open bite during rest (Condition 1).

Variable	Anterior open bite	No anterior open bite	*p*-Value
	Rank sum	Rank sum	
**Zone 1**			
Theta/beta	53.5	51.5	0.90*
Alpha/theta	68	37	0.05**
Alpha peak	71	34	0.02**
Beta	49	56	0.66*
Wavelength 2–38 Hz	57.5	47.5	0.52*
**Zone 2**			
Theta/beta	57.5	47.5	0.52*
Alpha/theta	60.5	44.5	0.31*
Alpha peak	65	40	0.11*
Beta	50	55	0.75*
Wavelength 2–38 Hz	57	48	0.565*

Zone 1, C3 (central sulcus area, left hemisphere). Zone 2, C4 (central sulcus area, right hemisphere). *p-value obtained with Student *T*-test. **p-value obtained with Mann–Whitney *U* test.

When comparing the data of children with and without AOB, no statistically significant differences are found for any variable in C2 (function) (Table 3).

**TABLE 3:** Comparison of cortex activity between children with and without anterior open bite during function (Condition 2).

Variable	Anterior open bite	No anterior open bite	*p*-Value
	Rank sum	Rank sum	
**Zone 1**			
Deglutition			
Theta/beta	49	56	0.65*
Alpha/theta	53.5	58.5	1.00*
Alpha peak	52	49	0.55*
Beta	49	56	0.43*
Wavelength 2–38 Hz	44.5	60.5	0.31*
Phonation			
Theta/beta	48.5	54.3	0.34*
Alpha/theta	54.2	53.5	0.83*
Alpha peak	46	49	0.46*
Beta	52	56	0.45*
Wavelength 2–38 Hz	53.5	60.5	0.21*
**Zone 2**			
Deglutition			
Theta/beta	54	61	0.33*
Alpha/theta	66	59	0.06*
Alpha peak	66	39	0.35*
Beta	49	56	0.55*
Wavelength 2–38 Hz	53.5	51.5	0.81*
Phonation			
Theta/beta	45	60	0.329*
Alpha/theta	66	39	0.083*
Alpha peak	66	39	0.084*
Beta	49	56	0.655*
Wavelength 2–38 Hz	53.5	51.5	0.898*

Condition 2, function (deglutition and phonation). Zone 1, C3 (central sulcus area, left hemisphere). Zone 2, C4 (central sulcus area, right hemisphere). *p-value obtained with Student *T*-test.

## Discussion

AOB is a complex malocclusion, often associated with functional alterations in the stomatognathic system. An improper tongue rest posture has been suggested as one of the primary contributing factors to AOB (Knösel et al., 2015). It is well established that the brain cortex, specifically the primary sensorimotor cortex, plays an important functional role in the regulation of tongue functions, such as deglutition and phonation (Kober et al., 2015; Ludlow, 2015). However, thus far, there have been no studies considering brain activation when the tongue is at rest or in function (deglutition and phonation) in children with AOB.

This exploratory investigation assessed the activation of the brain cortex activity of children with AOB at rest and during phonation and deglutition and evaluated the influence of the IQ, attention, and oxygen saturation on brain activity during those functions.

Efforts were made to use validated instruments and calibrated devices to assess IQ, attention, oxygen saturation, and brain activity and to test the influence of these variables on the brain cortex. Overall, the results of this investigation suggest that (1) IQ, attention, and oxygen saturation are not different between subjects with and without AOB and thus are not considered as confounding variables for the activity of the brain cortex in this study; (2) the activity of the brain cortex during rest was higher in subjects with AOB than with those with no AOB; and (3) the activity of the brain cortex during deglutition and phonation was not different between children with and without AOB.

Breathing disorders influence the cognitive and behavioral performance of children, including IQ and attention (Cazzolla et al., 2010; Lal et al., 2012). The present study did not demonstrate differences in oxygen saturation and TOVA between children with and without AOB. These findings disagree with the results of previous investigations (Blunden et al., 2000; Lal et al., 2012). Two major reasons for this disagreement must be analyzed. Firstly, children with mouth breathing or other breathing disorders were excluded from this exploratory study, in order to reduce the confounding variables that could affect the brain cortex activity as was previously demonstrated by other authors (Lee et al., 2019); and secondly, the results of the present investigation should be interpreted with caution, owing to the few subjects included in the sample, which could be considered a limitation of the study.

This was a study testing an original technique to evaluate brain activity in children with an AOB. As there are no data of previous investigations on the topic, it was recommended by the ethics committee of Universidad CES to test the new research hypothesis in a study with a pilot design in the first instance. The results of this hypothesis-generating study showed differences between the activity of brain cortex during rest, of children with and without AOB. Thus, obtained results are to be confirmed in a larger confirmatory study.

Differences in IQ and TOVA were not found between children with and without AOB. However, looking at the results yielded by the QEEG, higher alpha/theta activity was found, as well as a higher alpha peak during rest in children with AOB. These findings suggest possible differences in the brain cortex activity during cognitive demands (Usakli, 2010; Rodriguez-Larios and Alaerts, 2019), such as arithmetic tasks that were not measured with QEEG in this study. The correlation between the measurements of QEEG and clinical cognitive measurements in children with AOB deserves further attention for future investigations.

Regarding the effects of the tongue function on the anterior bite of children, several studies have measured forces of the tongue against the maxillary incisors and palate during rest and normal deglutition. The conclusions validated the hypothesis that the resting position of the tongue has a more significant role in the etiology of AOB than had deglutition and phonation functions (Shenoy et al., 2015). Furthermore, the cortical plasticity of individuals with incorrect tongue position during rest is different from the cortical plasticity of individuals whose tongue is in the palate during rest (Arima et al., 2011). The results of Arima et al. (2011) agree with those of this investigation, where an alteration in the sensorimotor cortex of subjects with AOB was found. In this context, therapies to establish correct rest position of the tongue, such as orofacial myofunctional therapy (OMT) (Van Dyck et al., 2016), could also influence the brain cortex plasticity, necessary to avoid relapse in orthodontic treatments of AOB. However, the quality of the evidence on this topic is still questionable (Koletsi et al., 2018).

When the function is analyzed, previous results demonstrated that during deglutition or phonation, incorrect position of the tongue can be observed in subjects with AOB. The reason could be a disturbed function of one or several afferent neurons (Maezawa et al., 2016). According to the results of the present study, there is no difference in brain activity during phonation and deglutition, between subjects with and without AOB (Shenoy et al., 2015).

Even when evaluating the activity of the left and right hemispheres during rest, deglutition, and phonation was not an objective of this investigation, it is interesting to mention the findings regarding this topic. Differences were found between the left and right hemispheres in both Condition 1 (rest) and Condition 2 (function). For most brain functions, the two hemispheres are a “mirror” of each other. However, there is evidence that shows that speech production is one of the most important lateralized features of the brain (Raemaekers et al., 2018). In the case of this pilot study, it was observed (even when not statistically compared) that differences were present at rest and in deglutition and phonation. This issue requires further exploration in other studies.

To our knowledge, this is the first study using brain mapping, which demonstrates that the brain activity in subjects with AOB is higher than in subjects without AOB at rest. A possible explanation could be the altered tongue posture during rest in AOB children. Future studies to test this hypothesis are suggested in larger samples, where the classification of the severity of the AOB could be assessed.

## Conclusion

The findings of this investigation provide insights about the cortex activity of the brain during the rest position of the tongue in children with AOB. This may mean an altered function, not only in the orofacial system but also in the central nervous system, that should be considered when diagnosing and treating AOB. Diagnosis of AOB should be focused not only on the teeth but also on the brain cortex.

## Data Availability Statement

The datasets generated for this study are available on request to the corresponding author.

## Ethics Statement

The studies involving human participants were reviewed and approved by the Ethics committee of Universidad CES. Written informed consent to participate in this study was provided by the participants’ legal guardian/next of kin.

## Author Contributions

CR and PB conceived the project. CR, PB, and KJ wrote the project. CR, PB, and DV were in charge of the control parameters. KJ and DV performed the measurements and collected the data. CR and PB were involved in planning and supervising the work. RM processed the data and performed the analysis. All authors interpreted the results and worked on the manuscript. All authors drafted the manuscript and designed the tables. All authors discussed the results and commented on the manuscript.

## Conflict of Interest

The authors declare that the research was conducted in the absence of any commercial or financial relationships that could be construed as a potential conflict of interest.
